# Chemical Structure Elucidation in the Development of Inorganic Drugs: Evidence from Ru-, Au-, As-, and Sb-based Medicines

**DOI:** 10.1002/ejic.202300717

**Published:** 2024-02-06

**Authors:** Alissa Lance-Byrne, Brent Lindquist-Kleissler, Timothy C. Johnstone

**Affiliations:** a Department of Molecular, Cell, and Developmental Biology University of California Santa Cruz Santa Cruz, California 95064, United States; b Department of Chemistry and Biochemistry University of California Santa Cruz Santa Cruz, California 95064, United States

**Keywords:** Inorganic chemistry, medicinal chemistry, bioinorganic chemistry, ruthenium, gold, arsenic, antimony

## Abstract

Structure elucidation plays a critical role across the landscape of medicinal chemistry, including medicinal inorganic chemistry. Herein, we discuss the importance of structure elucidation in drug development and then provide three vignettes that capture key instances of its relevance in the development of biologically active inorganic compounds. In the first, we describe the exploration of the biological activity of the trinuclear Ru compound called ruthenium red and the realization that this activity derived from a dinuclear impurity. We next explore the development of Au-based antitubercular and antiarthritic drugs, which features a key step whereby ligands were discovered to bind to Au through S atoms. The third exposition traces the development of As-based antiparasitic drugs, a key step of which was the realization that the reaction of arsenic acid and aniline does not produce an anilide of arsenic acid, as originally thought, but rather an amino arsonic acid. These case studies provide the motivation for an outlook in which the development of Sb-based antiparasitic drugs is described. Although antileishmanial pentavalent antimonial drugs remain in widespread use to this day, their chemical structures remain unknown.

## Graphical Abstract

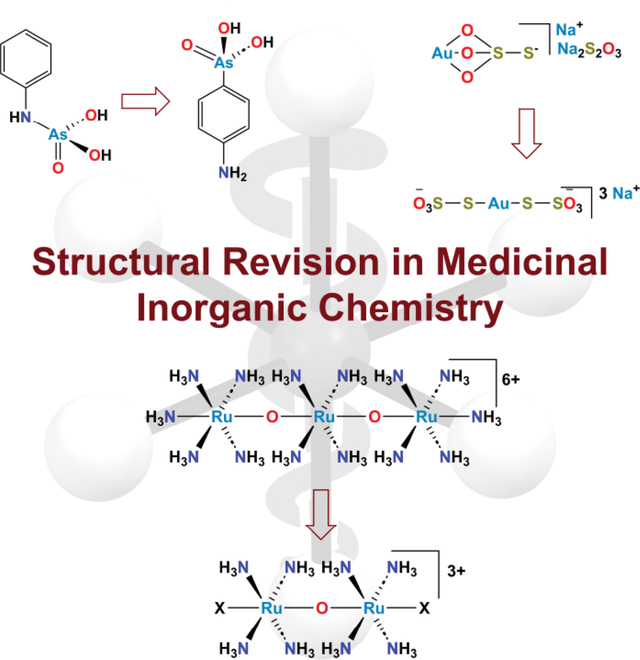

The importance of molecular structure elucidation in medicinal inorganic chemistry drug development is discussed and illustrated using three vignettes on Ru-, Au-, and As-based drugs. An outlook on Sb-based drugs is provided. The development of three classes of medicinal inorganic compounds is reviewed, highlighting the important role of structure elucidation. The progress of Ru-, Au-, and As-based compounds provide a justification for working to understand the structures of clinically used Sb-containing antiparasitic drugs.

## Introduction

The notion of structure permeates chemistry. The modern concept of a molecule is rooted in molecular structure, which comprises not only the three-dimensional arrangement of atoms, but also the graph representing the forces that bond these atoms together. The reasons for the importance of molecular structure vary from area to area within chemistry, but for the medicinal chemist aiming to develop new drugs, knowledge of molecular structure serves two primary functions: it provides a means of establishing purity and allows one to systematically alter the structure of a drug. Although inorganic chemists are frequently deeply interested in detailed bond metrics and the insight into molecular and electronic structure that they provide, in this review, we use *molecular structure* to simply indicate which atoms are bonded to which (atomic connectivity), as opposed to the precise three-dimensional coordinates of each atom in the molecule. Whereas the latter information is now most commonly obtained using single-crystal X-ray diffraction experiments, in the examples below, much of the contemporaneously actionable information about molecular structures was obtained using chemical methods as opposed to X-ray crystallography. To be clear, the biological activity of a molecule can certainly be exploited without its structure being understood – the medicinal properties of many molecules were enjoyed long before the notion of a molecule had even been conceived – but once that structure is uncovered, purity can be rigorously established and systematic molecular variation can be pursued.

We will begin this review with an overview of these two functions and the logic that underpins them. We will then provide a series of vignettes, each of which illustrates the transformative impact that a key structure elucidation played in the development of an area of medicinal inorganic chemistry: ruthenium-based mitochondrial calcium transport inhibitors, gold-based antitubercular and antiarthritic agents, and arsenic-based antitrypanosomal and antisyphilitic drugs. We then end with an outlook for the antimony-based drugs that are still widely used today to treat the neglected tropical disease leishmaniasis. Their chemical structures remain unknown and, with reference to the earlier vignettes, we describe the benefits that would come from closing this gap in knowledge.

## The roles of structure elucidation

**Figure 1.**
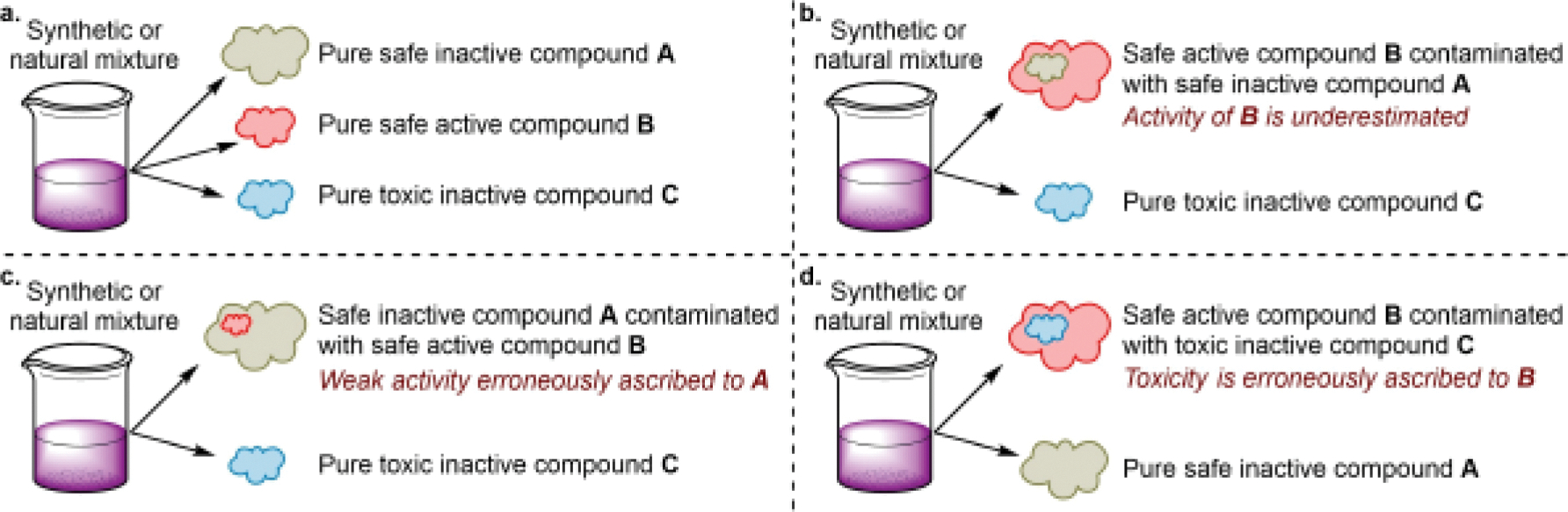
**a)** A synthetic or natural mixture containing a medicinally active compound can be separated into all of its components. **b)** The active component can be contaminated with inactive materials, leading to underestimated potency. **c)** An inactive component can be contaminated with an active species leading to activity being incorrectly assigned. This situation arises in the discussion of ruthenium red in Vignette 1. **d)** An active compound is contaminated with a toxic species. This situation may arise with the pentavalent antimonials, as described in the outlook.

The first, and perhaps most important, function that structure elucidation serves is in establishing the purity and composition of a biologically active substance. In a typical medicinal chemistry workflow, a pure compound is isolated from a reaction mixture or natural source and its biological activity is assessed (Figure 1a). Establishing the purity of the tested substance allows any observed activity to be ascribed to a specific molecule. Impure samples can give misleading results in several ways. Perhaps the most common situation arises when a substance that is thought to consist of only an active compound is contaminated with an inactive compound (Figure 1b), such as a simple benign salt or solvent, leading the activity per unit mass of the substance (and consequently the apparent activity per unit amount of the molecule) to be depressed and a potently active molecule to be overlooked in a subsequent activity screen. In an alternative framing of this same scenario, an inactive compound can be contaminated by an active compound, leading investigators to ascribe biological activity to the wrong molecule (Figure 1c). The discussion of ruthenium red (Vignette 1) will serve as an example of such a situation. In addition to activity/inactivity, impurities can confer avoidable toxicity on a sample (Figure 1d), and we highlight in the outlook that this may be the case for the antimony-based antileishmanial drugs.

It should be noted that, as with assessing biological activity, establishing purity does not require a knowledge of molecular structure. For example, although the temperature at which a molecular substance melts is intimately tied to its molecular (and crystal) structure, the *sharpness* of that melting point is dictated by the purity of that substance. Similarly, the specific retention time of a chromatographic signal, obtained for example by HPLC, is a function of molecular structure, but its *singularity* can be taken as a measure of purity. These examples notwithstanding, the modern means by which purity is established typically *do* require knowledge of the molecular structure of a substance. Based on the proposed structure of a molecule, the outcome of an analytical measurement is predicted, and numerical agreement between this prediction and an experimental measurement confirms the proposal. For example, a proposed molecular structure permits one to predict the % C, H, and N (or other elemental) composition. A combustion analysis that returns a composition consistent with the prediction within the uncertainty of the measurement is strong support that the substance is a pure sample of the molecule with the proposed empirical formula. This point may seem evident to a molecular inorganic audience, but we believe that it merits stressing that in the absence of a proposed molecular structure, this comparison is not possible. Although elemental analysis remains the gold standard in assessing small-molecule purity, many additional powerful and versatile methods in modern use can similarly corroborate the purity of a molecule with a given proposed molecular structure. Based on the proposed structure of a compound, for example, the number and type of signals expected from a particular spectroscopic experiment are predicted. The presence of all such signals helps to confirm the proposed identity of the compound, whereas the absence of any others provides strong support for the lack of spectroscopically active impurities. Of course, as the practicing chemist is aware, a disagreement between the proposed and experimental spectra can arise even with a pure sample if one’s understanding of the behavior or spectroscopic response of the molecule is deficient.

Structure elucidation secondly permits an archetypal medicinal chemistry exercise: systematic variation of the structure of a molecule and observation of the influence of those changes on a biological activity or physicochemical property. Most modern medicinal inorganic chemistry programs are designed in this tradition, such that the structure and purity of a molecule are determined after synthesis but before testing for activity. The activities of many classes of inorganic drugs, however, were discovered in the empirical tradition, whereby their biological activities were assessed before their molecular structures had been determined. The vignettes on gold- and arsenic-based drugs highlight that these classes both began in the empirical tradition, but that in each case a key structure elucidation allowed subsequent derivatives to be designed rationally. Indeed, the arsenic vignette describes what is arguably the first example of a modern medicinal chemistry research program in which an active molecular framework was systematically varied to discover compounds with improved activity. As in the case of purity, a knowledge of molecular structure is not needed to produce molecules of varying structure and assess their relative activities. It does, however, facilitate a *systematic* variation of that structure. It is interesting to consider how many historical inorganic drugs, both legitimately active ones and the products of immoral hucksterism, would not have been carried forward under our current drug development practices.

## Vignette 1: Ruthenium Red

**Figure 2.**
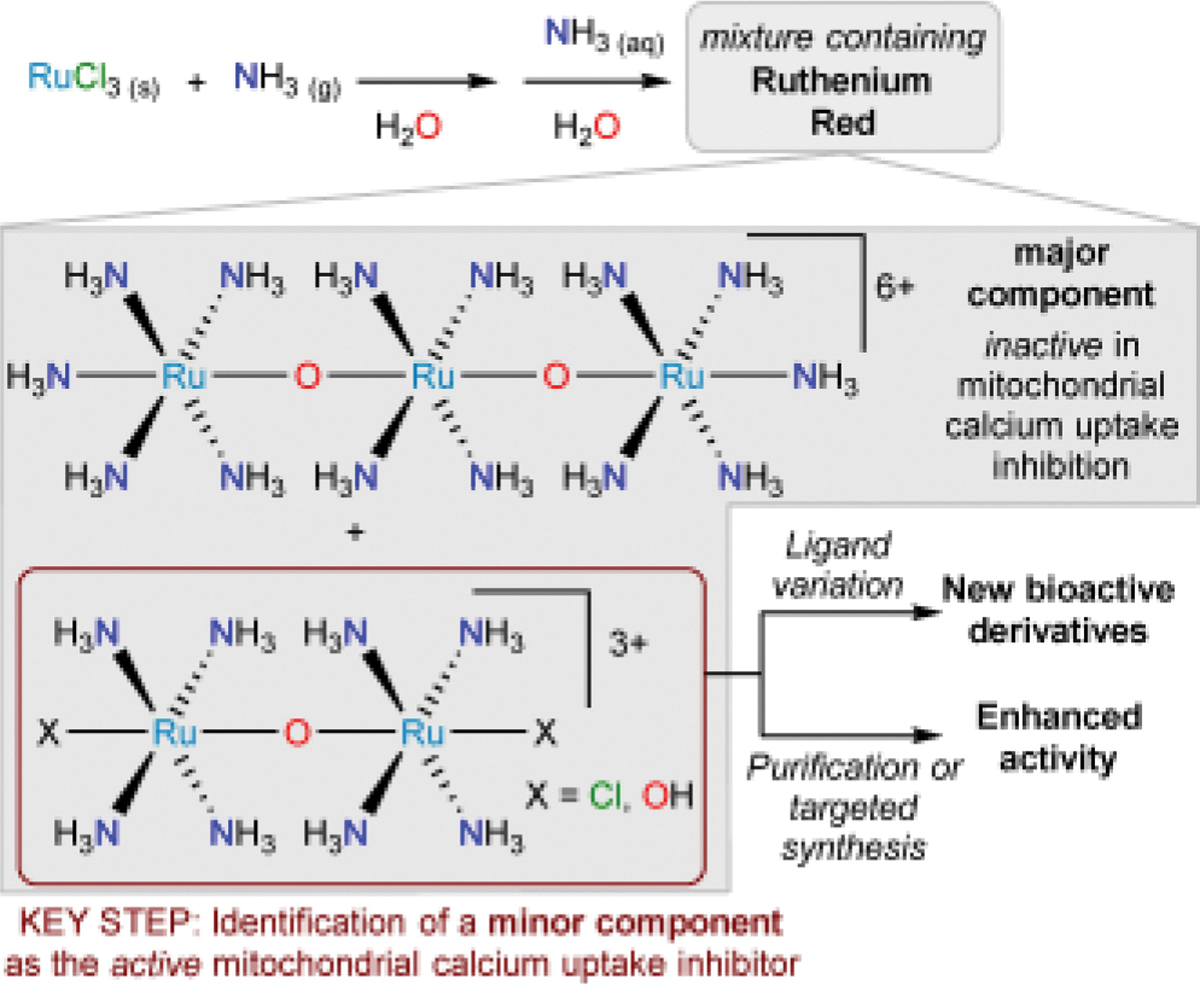
The preparation of ruthenium red and the description of this product as a mixture of species. A key step in understanding the chemistry and biology of this substance was the realization that the biological activity derived from an impurity formed during the synthesis. This realization allowed for enhanced activity to be obtained and for new bioactive derivatives to be discovered.

This vignette describes the discovery of multinuclear ruthenium compounds that can function as mitochondrial calcium transport inhibitors. This activity was initially ascribed to the trinuclear compound known as ruthenium red but was later found to stem from a dinuclear impurity (Figure 2). Elucidating the structure of the active species has allowed for more potent inhibitors to be produced and for new derivatives to be prepared and investigated.

In 1892, the reaction of ruthenium(III) chloride with dry ammonia, followed by dissolution in water and further reaction with aqueous ammonia, was reported to produce an intense red solution from which brown plates were isolated upon cooling.^[1]^ Redissolution of the brown plates recovered the red solution. This substance was found to be useful in staining plant microscopy samples, although the mechanism of this staining would not be uncovered for many years. It was in an 1893 paper describing its staining properties that the compound was first referred to as “rouge de ruthénium” (*ruthenium red*).^[2]^ Despite the fact that a second paper published that same year described its ability to also stain animal tissues and microorganisms,^[3]^ ruthenium red remained most popular as a stain for plant tissues for both light and electron microscopy.^[4–5]^ A dinuclear structure was originally proposed on the basis of elemental analysis.^[1]^ In 1936, a mononuclear Ru(III) proposal was put forward, but this was later refuted on multiple grounds.^[6]^ Perhaps the strongest criticism was that ruthenium red was at most weakly paramagnetic, but the mononuclear structure would be expected to give a paramagnetic compound with a room temperature magnetic moment near 1.73 BM (the μ_sO_ for a low-spin octahedral d^5^ complex). In 1961, an extensive study was released that shed light on many of the physical properties of ruthenium red: Faraday-method magnetic susceptibility measurements confirmed that the compound was only weakly paramagnetic (μ_eff_=0.77 BM per Ru atom), conductivity measurements showed the overall charge of the complex to be +6, and Ce(IV) redox titrations showed the average Ru oxidation state to be +3.33. These results led the authors to conclude that ruthenium red has a trinuclear structure featuring a Ru–O–Ru–O–Ru linkage.^[7]^ A molecular orbital theory description of the bonding in such a structure was proposed to account for the linear geometry, the stability of the complex, and the average Ru oxidation state.^[8–10] 99^Ru Mössbauer spectroscopy suggested the presence of Ru(III) and Ru(IV) in a 2:1 ratio, which agreed with the average Ru oxidation state of +3.33.^[11]^ In 1971, the crystal structure of a derivative obtained by reaction of ruthenium red with ethylenediamine was solved and confirmed the linear Ru_3_O_2_ motif.^[12]^ The structure of the NH_3_-bearing ruthenium red cation, crystallized as the thiosulfate salt, was ultimately solved in 1980 and confirmed the earlier work.^[13]^

At the time that the structure of ruthenium red was being investigated, electron microscopy experiments revealed that it effectively stained mitochondria, offering insight into the mechanism by which it stained plant and animal tissues.^[14–15]^ Mitochondria had recently been shown to accumulate Ca^2+^ ions,^[16–17]^ although it should be noted that those seminal discoveries took place within the context of earlier work on the topic.^[18]^ These Ca^2+^ ions were, in turn, known to bind mucopolysaccharides. Because ruthenium red was proposed to stain tissues by interacting with mucopolysaccharides, it was tested for its ability to impact mitochondrial Ca^2+^ transport.^[19]^ After the Ca^2+^ transport inhibitory properties of ruthenium red were established,^[19–20]^ it was observed that unpurified commercial samples were actually *more* effective at inhibiting Ca^2+^ transport than purified material.^[21–22]^ It is noteworthy that the unpurified, but more active, ruthenium red investigated in this work had an additional UV-visible absorption band near 355 nm that was absent in the purified ruthenium red.^[22]^ The presence of impurities in commercial ruthenium red has long been appreciated in the context of both microscopy^[4]^ and pharmacology^[23–24]^ studies.

**Figure 3.**
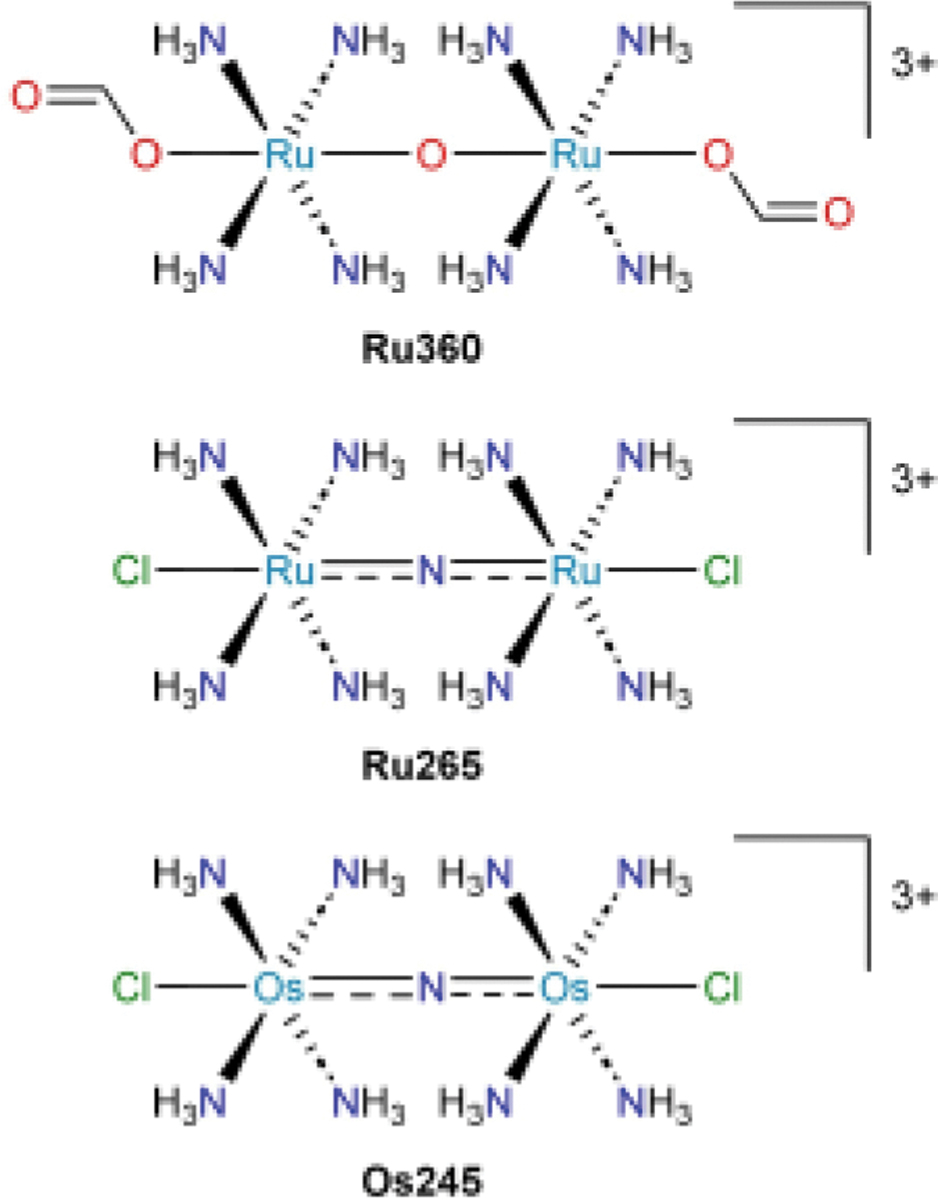
Biologically active compounds discovered as a result of the investigation into the molecular identity of the active component of ruthenium red.

It wasn’t until two decades later, in 1991, that the active agent was determined to be a dinuclear oxo-bridged Ru compound with an average Ru oxidation state of +3.5.^[25–26]^ The dinuclear species was isolated from the ruthenium red synthetic reaction mixture by ion exchange chromatography. Because ammonium formate was used as the eluent, the complex was isolated with capping formate ligands and had the composition [(μ-O)((HCO_2_)(NH_3_)_4_Ru)_2_]Cl_3_. The compound features a UV-visible absorption signal at 360 nm, so it was dubbed *Ru360* (Figure 3). Armed with the knowledge of the structure of the active agent, it was possible for medicinal inorganic chemists to reproducibly confirm Ru360 activity,^[27]^ to probe its mechanism of action,^[28–31]^ to develop improved syntheses of the multinuclear pharmacophore,^[32–34]^ and to derivatize this scaffold to improve activity (Figure 3).^[35–41]^ These developments, including the extension to Os complexes, have been reviewed recently.^[42–44]^ It is currently suspected that these compounds function by inhibiting the mitochondrial calcium uniporter, but efforts to continue to elucidate their mechanism of action are ongoing.^[42]^

The story of ruthenium red provides a prototypical example of a substance that was erroneously thought to be active, when the activity was in fact conferred by a small amount of a contaminant (Figure 1c). The active component, which is related to Ru360, could be chromatographically separated from ruthenium red without needing information about its structure, but that structure elucidation ensured that the active compound had indeed been obtained cleanly. Moreover, knowledge of the molecular structure of Ru360 allowed for systematic derivatization of the framework to greatly enhance the Ca^2+^ transport inhibition.

## Vignette 2: Gold-based antiarthritic agents

**Figure 4.**
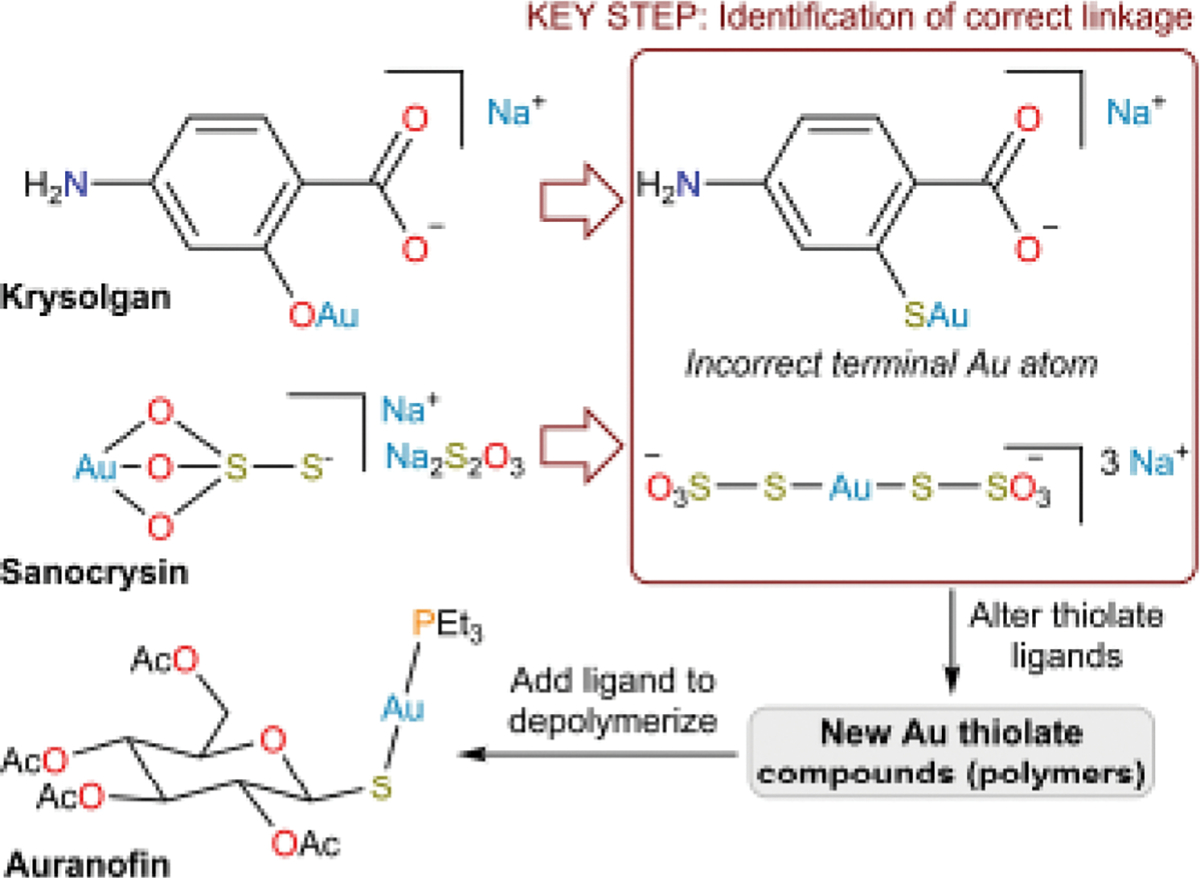
The conceptual development of Au-based antitubercular and antiarthritic drugs. A key step in this progression was the realization that ligands were attached to the Au centers through S atoms. As described in the main text, historical formulations of Au(I) complexes had depicted them with terminal Au atoms.

This vignette provides a brief introduction into the use of gold-based medicines in the modern era (19^th^ century to present) to highlight the role that a key set of structure elucidations played in the development of a novel antiarthritic drug. In early medicinal Au compounds with ligands bearing multiple possible donor atoms, O atoms were frequently proposed to bind Au(I) preferentially over S atoms. Understanding that these ligands in fact bind through their S atoms was critical in establishing the way the ligands should be systematically varied, which ultimately led to the discovery of the antiarthritic drug auranofin (Figure 4). Research into the use of Au complexes for multiple indications remains an active area of investigation.^[45,168]^

**Figure 5.**
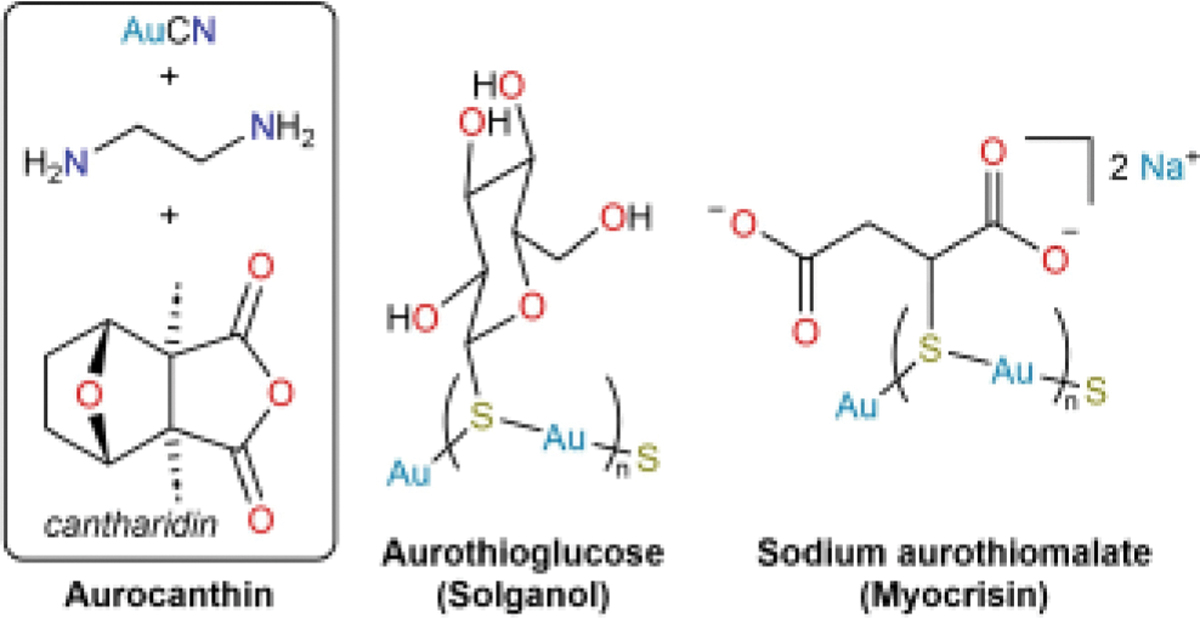
Biologically active Au complexes, highlighting their polymeric nature. The structure of aurocanthin was never determined, and so the constituent components are shown.

Because of its high ionization potential, Au can occur in nature in its elemental form. As such, it was one of the early metallic elements known to the ancients, and elemental Au has consequently long been used to treat a variety of illnesses. In the early 19^th^ century, for example, rubbing finely divided Au into the gums was used as a treatment for syphilis,^[46]^ but even at that time this treatment was scathingly criticized.^[47]^ At around the time that ruthenium red was discovered (*vide supra*), it was observed that gold(I) cyanide showed some *in vitro* efficacy in the treatment of tuberculosis.^[48]^ In the early 20^th^ century, it was claimed to have a significant effect in human patients.^[49]^ This result launched the era of chrysotherapy (also known as krysotherapy and crysotherapy; from the ancient Greek χρυσός meaning “gold”). In 1913, a compound called *aurocanthin* was prepared by combining gold(I) cyanide with the product of the reaction of ethylenediamine with cantharidin, a potent vesicant isolated from blister beetles (Figure 5).^[50]^ The cantharidin was intended to function as a carrier for the Au and the ethylenediamine was introduced to attenuate the toxicity of the cantharidin. In 1917, the same author described a compound called Krysolgan, which was described as “das Natronsalz einer komplexen 4-Amino-2-aurophenol-1-carbonsäure,” [the sodium salt of a complex 4-amino-2-aurophenol-1carboxylic acid].^[51]^ The synthesis was not described in detail and no characterization data were provided, but the “aurophenol” terminology suggests an ArO–Au linkage (*vide infra*). The compound was referred to as 4-amino-2-aurophenol-1-carbonsäure in medical papers from that time, and the 1925 E. Merck Annual Report described Krysolgan as “p-amino-o-aurophenol carbonic acid in which the gold is indirectly attached to the benzol nucleus in a complex manner.”^[52]^ It was not until the mid-1920s that a review paper presented the structure of Krysolgan as a thiolate complex featuring an ArS–Au linkage (Figure 4).^[53]^ Interestingly, an unexplained parenthetical question mark was placed next to the label for the ArS–Au structure in this paper, potentially alluding to the discrepancy with the earlier description. It is worth noting that because the original report of Krysolgan appeared in the German literature during World War I, there may have been limited communication of these ideas to readers outside of Germany, such as the British author of the aforementioned review.^[53]^ The following year, the original discoverer of Krysolgan published a paper in which the compound was described as “4-Amino-2-Auro*merkapto*benzol-1-Carbonsaures Natrium” (italics added) and a line diagram was provided that confirmed the proposed ArS–Au linkage.^[54]^ No comment was made about the earlier formulation, so it is unclear if this change comprised a simple correction, a change in the nature of the material itself, or reflected a revision of the investigators’ understanding of the structure of the compound. We favor the latter interpretation because in the paper, the authors claimed that Krysolgan was the first gold thiolate complex to be discovered [“Damit war die erste organische Komplexverbindung des Goldes gefunden.”], although we note that in the 1834 report of the first synthesis of ethanethiol, a description is given of its reaction with gold(III) chloride to yield a white solid that is likely [Au(SEt)]_n*_^[55]^

In the 1920s, sodium aurothiosulfate, which had been synthesized in the mid 1800s,^[56]^ began to be used as an antitubercular agent under the name Sanocrysin (Figure 4).^[57]^ In the early stages of its medicinal use, sodium aurothiosulfate was described as [(AuS_2_O_3_)Na_1_,Na_2_S_2_O_3_] and was proposed to release [AuS_2_O_3_]^−^ upon dissolution in water.^[57]^ A 1926 description of an improved synthesis of sodium aurothiosulfate further proposed that a [AuS_2_O_3_]^−^ complex anion released upon dissolution existed in an equilibrium between O-bound and S-bound linkage isomers.^[58]^

The structures proposed for Krysolgan, Sanocrysin, and other gold complexes typically featured Au(I) centers with terminal, one-coordinate binding modes. RSAu, ROAu, and [O_3_SSAu]^−^ were written in the same manner that RSNa, ROK, or [O_3_SSH]^−^ might be written now (Figure 4). Although in current transition-metal parlance, “valence” and “oxidation state” are frequently used interchangeably (although not without justifiable contest),^[59]^ in the late 19^th^ and early 20^th^ centuries, valence reflected the combining power of an atom. A monovalent Au(I) center would be expected to make only one bond and molecular structures were proposed accordingly. Crystallographic and ebullioscopic studies in the late 1930s ultimately revealed that Au(I) complexes typically assume linear 2-coordinate geometries.^[60]^ This reasoning was subsequently applied to the structure of aurothiosulfate, suggesting that it forms an S-bound, linear, 2-coordinate complex.^[61]^ This structure was ultimately later confirmed using Mössbauer spectroscopy,^[62–63]^ X-ray crystallography,^[64–65]^ and EXAFS.^[66]^

With the RS–Au link of active gold complexes established, investigators engaged in a systematic variation of the R group to improve upon antitubercular activity. At the time that this search for improved treatments of tuberculosis was underway, a proposal was put forward that rheumatoid arthritis was similarly caused by infection with a microorganism.^[67]^ Although rheumatoid arthritis is currently understood not to arise via this mechanism, many Au compounds were nonetheless found to be effective antiarthritic agents.^[68]^ Even at the time, it was appreciated that these compounds were active *despite* their basis in an incorrect etiology.^[69]^ One of the Au compounds that rose to prominence as an antiarthritic was aurothioglucose (Solganol).^[70]^ It was later appreciated that Au(I) thiolate complexes like aurothioglucose and sodium aurothiomalate (Myocrisin) form oligomers (Figure 5).^[71–72]^ In an effort to increase the bioavailability of orally administered Au-based antiarthritic agents, the framework was further derivatized. In particular, the inclusion of lipophilic phosphine ligands was explored, ultimately leading to the preparation of a Au(I) complex with one triethylphosphine ligand and one per-*O*-acetylated thioglucose, bound as a thiolate.^[73]^ This complex, known as *auranofin* (Figure 4), remains in clinical use to this day to treat arthritis, though we note that it is not used to treat tuberculosis, which is now treated with a regimen of organic drugs.^[45]^

This vignette began with the empirically discovered antitubercular activity of simple gold salts, which were subsequently combined with compounds (cantharidin, aromatic amino acids, thiosulfate) to increase their parasite-targeting ability or decrease host toxicity. Once the molecular structures of these drugs were understood, they could be systematically derivatized in a rational way to improve antitubercular or antiarthritic activity. This research ultimately led to the development of auranofin, one of the more widely employed metal-based therapeutics of the modern era.

## Vignette 3: Arsenic-based antiparasitic drugs

**Figure 6.**
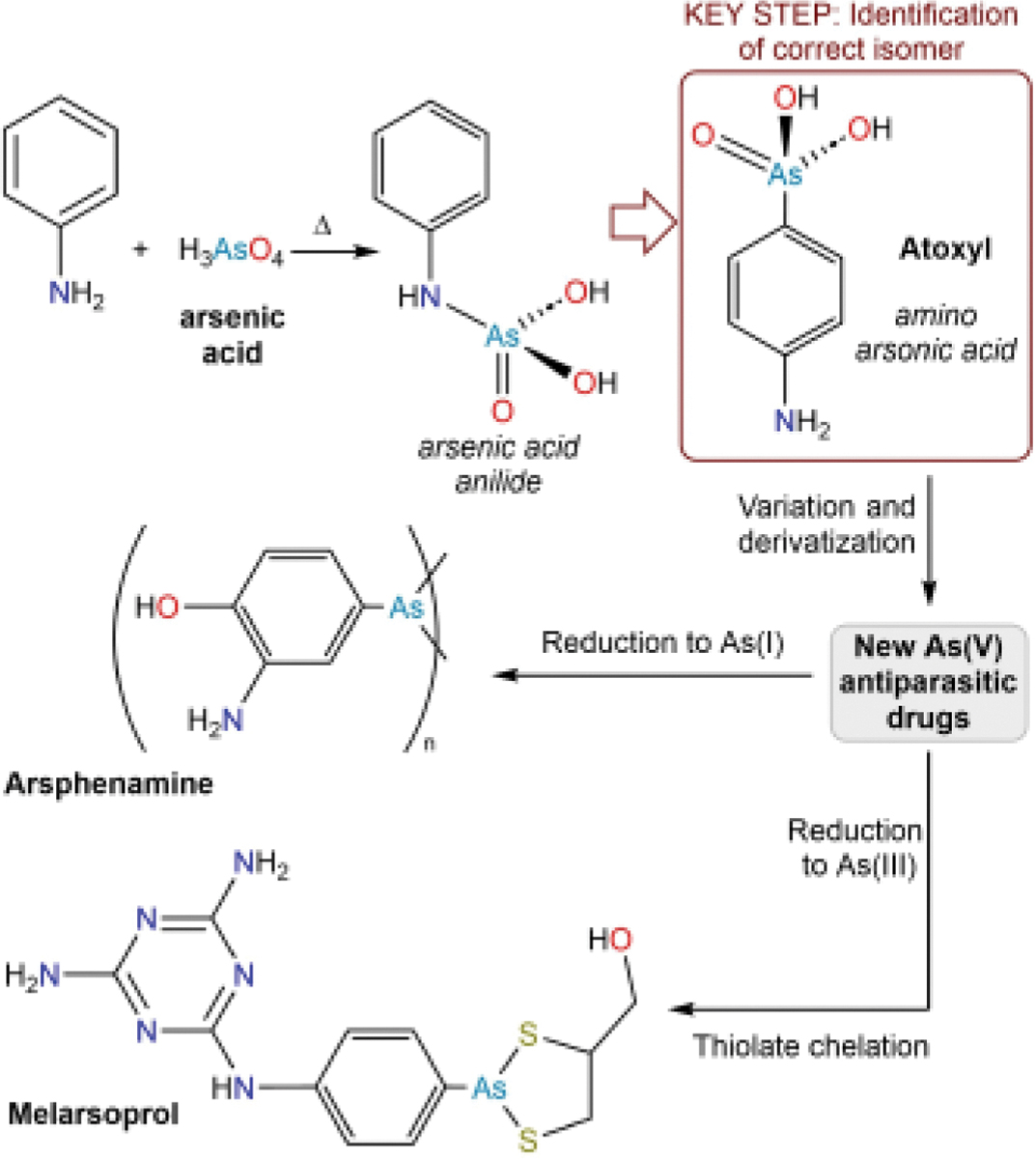
The development of As-based antiparasitic drugs. A key step in the development of these compounds was the realization that atoxyl was not an arsenic acid anilide, but rather an amino arsonic acid. This realization permitted systematic derivatization at the amino group and redox chemistry at the As center.

This vignette describes what is arguably the first example of a modern medicinal chemistry research program in which an initially active compound is systematically varied in structure to improve upon biological activity. The species under investigation were arylarsonic acids with antitrypanosomal and antisyphilitic activities. This systematic variation was rooted in the elucidation of the molecular structure of an arsenic compound that was dubbed atoxyl. The knowledge that this compound bore a free aromatic amine group provided a means of systematically preparing derivatives, which ultimately led to the discovery of the antisyphilitic drug arsphenamine and the antitrypanosomal drug melarsoprol (Figure 6).

In the 19^th^ century, it was reported that grains of arsenic could be used to treat animals infected with trypanosomiasis.^[74–76]^ Although it is not specified in the original reports, we suspect that the administered material was likely an arsenate or arsenite salt. At around this same time, fundamental investigations in the budding field of organic chemistry were being performed on aniline with the goal of generating new dyestuffs.^[77]^ The reaction of arsenic acid (H_3_AsO_4_) with aniline expectedly forms the anilinium arsenate,^[78]^ but it was discovered that heating this salt yielded a new compound, which was presumed to be the anilide of arsenic acid, C_6_H_5_NHAs(O)(OH)_2_ (Figure 6).^[79]^

By the beginning of the 20^th^ century, simple inorganic arsenate salts were still among the most effective treatments for the infectious tropical disease trypanosomiasis, but they were plagued by high toxicity. In an effort to mitigate this toxicity while maintaining activity, organoarsenic compounds were tested, including the presumed arsenic acid anilide described above.^[80–81]^ Although still a highly effective antitrypanosomal agent, this compound had a sufficiently reduced number of toxic side effects that it was called *atoxyl*.^[80]^ We note that, however attenuated its toxicity might be, atoxyl still gave rise to a multitude of undesired effects (*vide infra*).

The reactivity of atoxyl, particularly its stability to acids and bases, did not agree with the reactivity known for other anilides, and in 1907 it was proposed that the As atom was attached directly to the aryl ring.^[82]^ That same year, the compound was identified as *para*-aminophenylarsonic acid (arsanilic acid) through a series of classical reactivity tests (Figure 6).^[83–84]^ In addition to the inability to hydrolytically produce aniline, the compound readily underwent acetylation, diazotization, and condensation reactions, as expected for a species with a free amine. Moreover, reaction with hydroiodic acid resulted in clean elimination of arsenic acid and installation of an I atom onto the aromatic ring. The *para*-substitution pattern of arsanilic acid was confirmed because, in this reaction with hydroiodic acid, only *para*-iodoaniline was obtained. The importance of this structural determination for the field of medicinal chemistry cannot be overstated. Having established the presence of a free amine in arsanilic acid, this group was used to systematically derivatize the compound, and these derivatives were tested for trypanocidal activity. We will discuss these trypanocidal efforts below, but we will first describe another important development in infectious disease medicine that occurred around this time.

In 1905, the causative agent of syphilis was determined to be a bacterial spirochete.^[85]^ This discovery led investigators to propose that syphilis might be amenable to treatment using the types of antiparasitic drugs that had proven effective against trypanosomiasis. Early studies showed that arsanilic acid was indeed effective but exhibited undesired side effects.^[86]^ To find yet further improved drugs, a research program was launched in which the arsanilic acid framework was systematically varied in terms of its molecular structure and antisyphilitic activity was recorded as a function of these variations. Because of the consideration that was given to synthesis of molecules of specified targeted structures, this endeavor can justifiably be viewed as the advent of modern medicinal chemistry. The effort was led by Paul Ehrlich and in his 1908 Nobel Lecture,^[87]^ he articulates the rationale behind a key component of this research program. It had been observed both that As(V) compounds were generally less toxic than As(III) compounds and that As(V) compounds were capable of being reduced to As (III) following administration. It was further proposed that As(V) compounds themselves did not have antiparasitic activity, but rather only exhibited activity upon reduction to As (III) in the biological system under investigation. Ehrlich’s research program therefore expanded from the then-popular As(V) species to include As(III) and As(I) compounds.^[88]^ The well-celebrated outcome of this program was the discovery of *arsphenamine* (marketed as Salvarsan).^[89]^ Arsphenamine was the As(I) product obtained from the reduction of 3-amino-4-hydroxyphenylarsonic acid. Until the discovery of penicillin, arsphenamine and its derivatives were the frontline therapies for syphilis in many parts of the world.^[90]^ The development of arsphenamine would not have been possible without the realization that the parent compound, arsanilic acid, was an amino-substituted arylarsonic acid (Figure 6).

**Figure 7.**
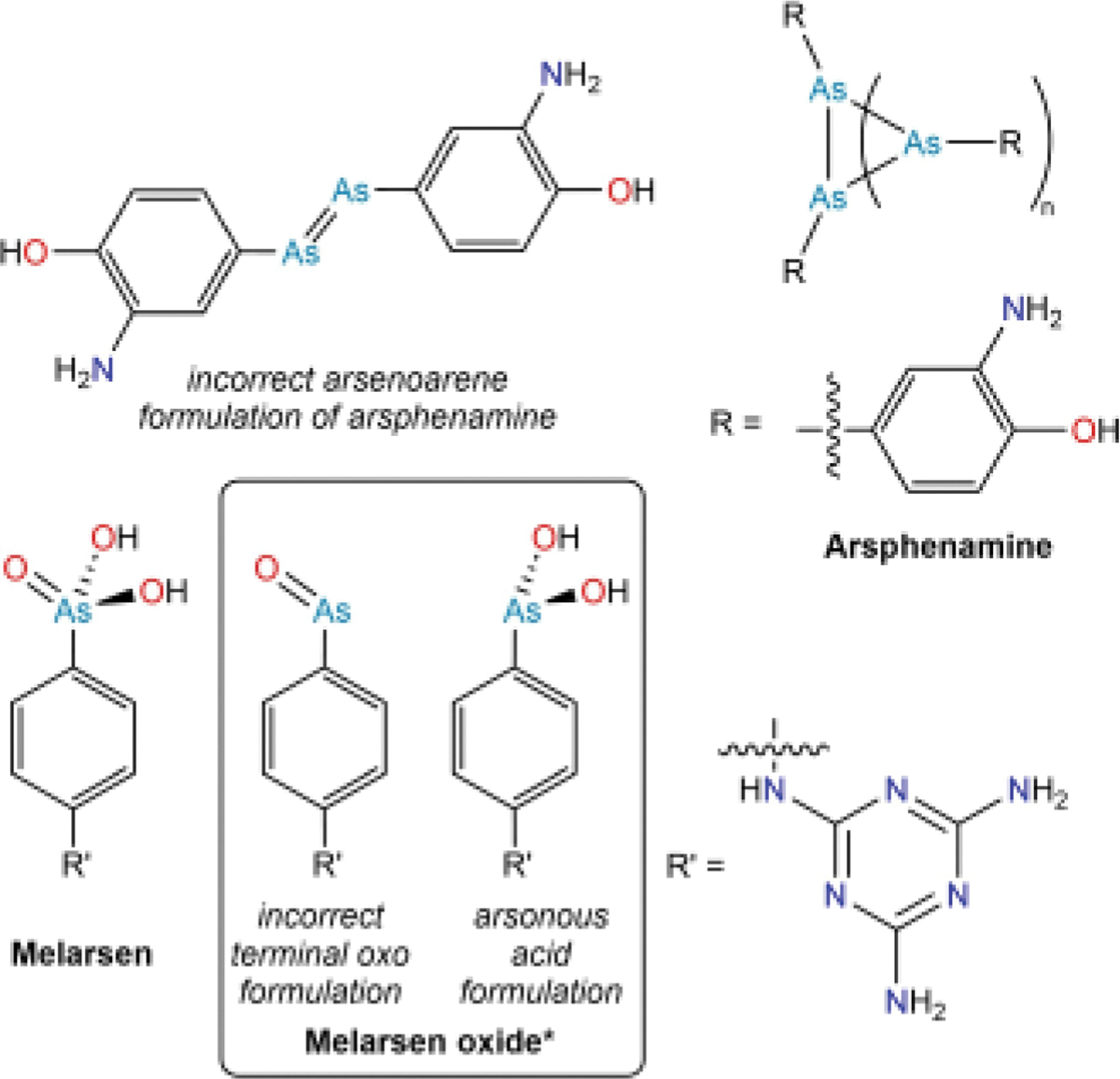
Some of the biologically active As compounds developed after the elucidation of the structure of atoxyl. *We acknowledge that the historically used name *melarsen oxide* can be particularly confusing given that it is formally the result of *removing* an oxide from melarsen.

Given the structural focus of this review, it is interesting to note that arsphenamine was initially proposed to be an arsenobenzene derivative with an As═As double bond in analogy to diazobenzene compounds (Figure 7).^[89]^ Although second-row elements are able to form strong double and triple bonds because of efficient π-symmetry overlap between p orbitals of adjacent atoms, this overlap decreases substantially for elements of greater atomic number. Indeed, compounds featuring As═As bonds are typically highly unstable, and it was not until the 1980s that the first example of an isolable compound featuring a genuine unsupported As═As bond was reported.^[91]^ Following the discovery of arsphenamine, simple alkylarsenic(I) and arylarsenic(I) compounds were subsequently shown to assume oligomeric structures featuring –As–(As)_*n*_–As chains or cycles.^[92–95]^ It was only in 2005 that such an oligomeric structure was confirmed for arsphenamine using electrospray ionization mass spectrometry.^[96]^ Unlike the distinction between the arsonic anilide and arsonic acid formulations of arsanilic acid described above, the systematic variation of arsphenamine derivatives would not have been significantly impacted by whether they existed as doubly-bonded dimers or higher-order oligomers; even had they been formulated as oligomers in the early 1900s, the synthetic reactions to target them (e.g., variations of substitution patterns on the aromatic ring and functionalization of the amine group) would have been the same.

During the rise of arsphenamine as an antisyphilitic drug, research continued into arsenic-based antitrypanosomal agents. In 1940, *p*-(2,4-diamino-*s*-triazinyl-6)-aminophenylarsonic acid was found to have further reduced toxicity as compared to arsanilic acid, while maintaining activity in cases of human African trypanosomiasis.^[97–98]^ The compound could alternatively be named 4-melaminylphenylarsonic acid and came to be called *melarsen* (Figure 7). Following the same rationale that led investigators to study reduced compounds such as arsphenamine, reduced forms of melarsen were explored. The most commonly studied reduced form was typically depicted with a two-coordinate As(III) center singly bonded to the aryl group and doubly bonded to a terminal oxo group. Such a compound can be called *p*-(2,4-diamino-*s*-triazinyl-6)-aminophenylarsenoxide, so the substance was dubbed *melarsen* oxide (Figure 7).^[99]^ We note that because of the instability of terminal oxo compounds of heavy pnictogens,^[100–103]^ these species would exist either as self-associated oligomers or as hydrated arylarsonous acid derivatives. By the late 1940s, melarsen oxide had been observed to rapidly clear trypanosomes from the blood, but it was still plagued by side effects.^[99]^

To further improve the safety profile of melarsen derivatives, use was made of chemistry that had been developed during World War II. Synthetic chemical warfare agents had been under intense investigation since the previous World War, and the product of the addition of trichloroarsine across acetylene, chlorovinyldichloroarsine (also known as lewisite), had been discovered to act as a vesicant.^[104]^ It had been applied as a chemical warfare agent in World War II, and consequently an extensive international research program was launched to identify an antidote for lewisite. Much of the progress appeared in government reports or was classified, but the program was contemporaneously reviewed at the end of the war.^[105–106]^ Key to this endeavor was earlier work that had revealed that As(III) species could be rendered less toxic through reaction with thiols, including chelating dithiols.^[107]^ The lewisite antidote research program ultimately identified 2,3-dimercaptopropanol as the most promising agent and it came to be known as British Anti-Lewisite or BAL. Capitalizing on the extensive amount of work performed on 2,3-dimercaptopropanol, it was tested as a passivating agent for melarsen oxide. Although 2,3-dimercaptopropanol was the specific dithiol used to develop an improved form of melarsen because of the lewisite work, the general motivation for introducing sulfur-containing substituents into medicinally active arsenicals stemmed from the increased tolerance for melarsen exhibited by patients eating Swiss cheese and the sulfur compounds it contains.^[108]^ The compound formed by the combination of 2,3-dimercaptopropanol (BAL) and melarsen oxide was initially dubbed Mel B,^[109]^ and later came to be known as *melarsoprol* (Figure 6). Despite its side effects, melarsoprol continues to be used to this day to treat severe late-stage gambiense human African trypanosomiasis.^[110–111]^ As noted above, As-containing drugs have been formulated as As(V),As(III), and As(I) compounds, and with or without passivating ligands. The active agent is typically taken to be an As (III) species bearing exchangeable hydroxyl substituents (e.g., the arsonous acid depicted in Figure 7). Redox speciation and substituent exchange are, therefore, key players in the behavior of these drugs.

## Outlook: Pentavalent antimonials

**Figure 8.**
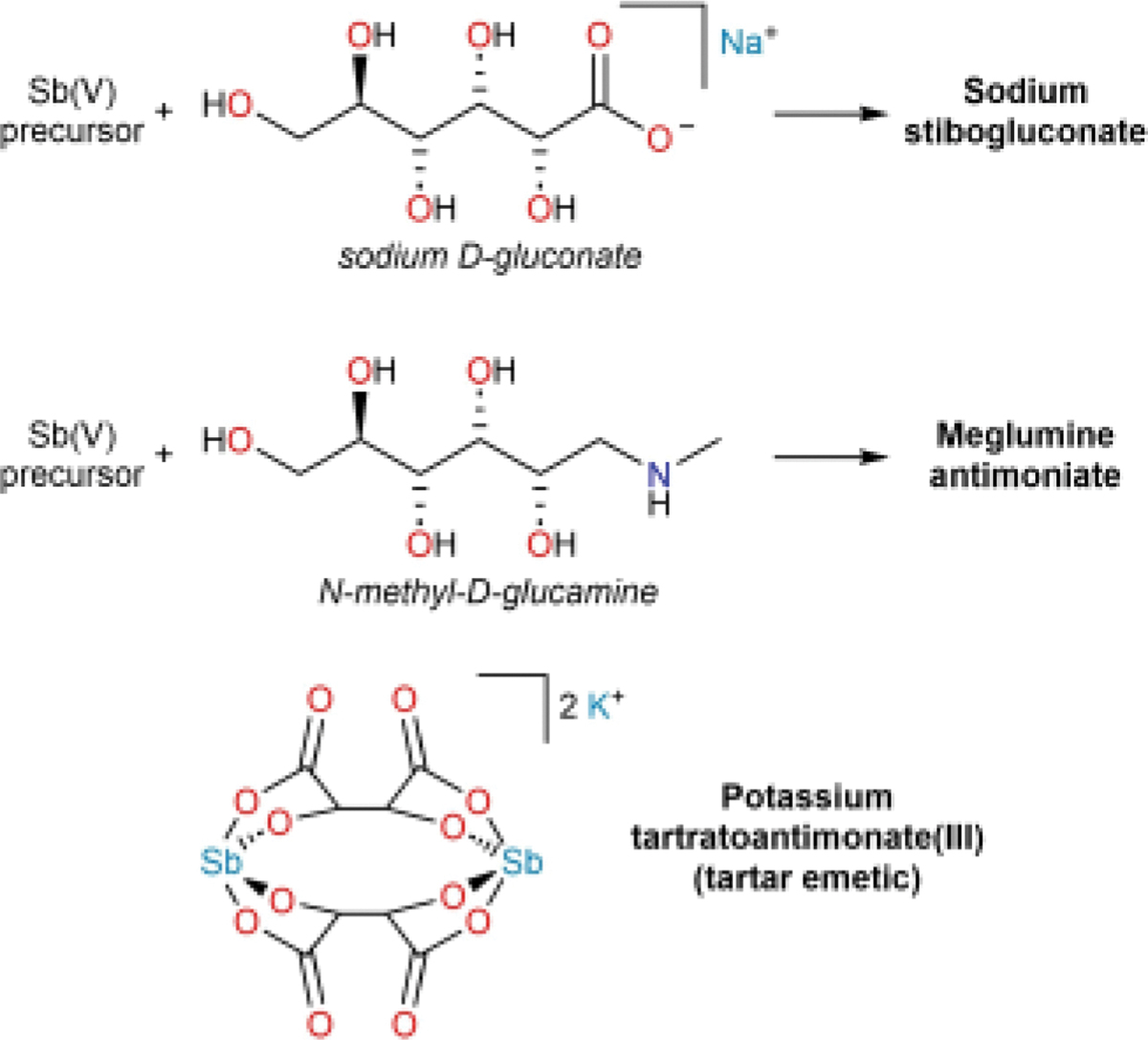
Biologically active Sb-based drugs. The structures of sodium stibogluconate and meglumine antimoniate are not depicted because they remain unknown.

This outlook comprises a final vignette focused on Sb-containing drugs and the development of the pentavalent antimonials that remain in use today to treat leishmaniasis: sodium stibogluconate and meglumine antimoniate (Figure 8). Unlike the drugs in the previous vignettes, however, the structures of these drugs remain unknown. A case is made that understanding their structures could afford advantages similar to those described for the other classes of compounds discussed in this review.

While the antitrypanosomal action of As-containing compounds was being explored in the early 20^th^ century, investigators also targeted Sb-containing compounds with the expectation that, as members of the same family on the periodic table, they might exhibit similar biological activity, and identified sodium antimony tartrate as a candidate antitrypanosomal drug^.[112–116]^ The compound had first been reported in 1842,^[117]^ but its synthesis and characterization were revisited in 1908.^[118]^ No molecular structure was proposed, but on the basis of gravimetric analysis, the crystalline substance isolated and tested was formulated as the hydrate of the tartrate double salt. This compound was a direct analog of potassium antimony tartrate, also known as tartar emetic, which, for better or worse, has held a longstanding place in the history of medicine.^[119]^ The ability to recrystallize antimony tartrate complexes ensured that substances of reproducible purity were used, which consistently afforded an empirical formula in which Sb and tartrate were present in a 1:1 ratio. Much later X-ray crystallographic studies revealed that these compounds featured a common tartratoantimonate(III) complex anion, which forms with a 2:2 stoichiometry (Figure 8).^[120]^

At the time that the antitrypanosomal activity of antimony compounds was being explored, groundbreaking discoveries in another tropical disease were being made. In 1903, W. B. Leishman and C. Donovan documented the observation of a microorganism^[121–125]^ that was named *Leishmania donovani* that same year.^[126]^ This microorganism was found to be the causative agent of a class of diseases from South Asia, Africa, and the Mediterranean that are now collectively called leishmaniasis.^[127–128]^ The disease, as it presented in India, was frequently called kala-azar, and this name was often extended to diseases caused by other types of infections with *Leishmania* spp. In 1909, it was discovered that there were diseases endemic to the Americas that were also caused by leishmanial parasites.^[129–130]^ By the end of the first decade of the 20^th^ century, arsenical treatments, including arsanilic acid, had been used with varying levels of apparent success in the treatment of both Old World and New World leishmaniasis.^[131–135]^

In 1913, the first report appeared of the ability of an Sb-containing compound to treat leishmaniasis; it described the use of tartar emetic to treat the disease in Brazil.^[136]^ Tartar emetic was then successfully used to treat leishmaniasis in Italy and India.^[137–138]^ The initially promising results obtained with tartar emetic led to the investigation of organoantimonials, in analogy to the organoarsenicals that proved to be active against trypanosomes. Among the first to be prepared was the antimony analog of arsanilic acid, which was described as “*para*-aminophenylstibinic acid” (although it would now be called *para*-aminophenylstibonic acid) and given the moniker stibamine.^[139]^ Although the compound was initially described as a monomeric stibonic acid, it is now known that, like arylstibine oxides,^[101–103,140–142]^ arylstibonic acids can self-associate into higher-nuclearity structures.^[143–146]^ In the absence of oligomerization, they may also remain monomeric but expand their coordination sphere, possibly with ligand exchange occurring.^[147]^ As a result, the molecular structure of stibamine remains unknown.

In the 1920s, a significant advancement came from the combination of stibamine with urea to give a substance with potent antileishmanial activity that was called *urea stibamine*.^[148–151]^ As an alternative to reaction with urea, stibamine was also allowed to react with glucose to afford a substance that was described as a glucoside of stibamine and marketed as Neostam;^[152]^ it exhibited high aqueous solubility and reduced toxicity.^[153–154]^ In addition to the considerations raised above for stibamine, understanding of these derivatives was further complicated by the lack of systematically applied purification procedures, likely because of the inability to readily conduct recrystallizations. The procedures used to prepare these substances were ill-defined and their molecular structures remain debated.^[155–159]^ Even in the face of this uncertainty and variability, however, they unquestionably played a significant role in the management of leishmaniasis in India in the first half of the 20^th^ century.^[152,160]^

The strategy of using sugar derivatives to increase solubility was explored further in Germany in the 1930s and led to the production of *Solustibosan*, a complex of gluconic acid (described as hexonic acid) and Sb(V).^[161]^ Solustibosan was commercialized by Bayer, but international access to this drug was limited during World War II. To address this issue, the Wellcome Chemical Research Laboratories in Britain launched a research program to develop additional antileishmanial drugs and produced *Pentostam*.^[162–163]^ It was later realized that Solustibosan and Pentostam were prepared via the same process, and the resulting substance came to be known generically as sodium stibogluconate.^[163]^ The enhanced solubility and efficacy of this drug led to its widespread adoption. Like urea stibamine, however, it was not subjected to typical purification procedures, and variations in the synthetic procedure would lead to products of inferior quality that exhibited reduced activity and greater toxicity.^[163]^ In response to the shortage of Solustibosan during the war, France also secured the domestic production of a pentavalent antimonial drug. Spécia, a subsidiary of Rhône-Poulenc, produced meglumine antimoniate, a complex of Sb(V) and N-methylglucamine, and marketed it as Glucantime.^[164]^ It is interesting to note that although sodium stibogluconate was disseminated more widely around the world, meglumine antimoniate became one of the primary treatments of the New World leishmaniasis endemic to Latin America. The Société chimiques des usines du Rhône, which became the company that would develop Glucantime, had established the Brazillian subsidiary Companhia Química Rhodia Brasileira in São Paulo in 1919. By the middle of the 20^th^ century, the Brazilian group had become one of Rhône-Poulenc’s largest pharmaceutical factories outside of France. The ready supply of meglumine antimoniate that it offered led to this drug being more readily adopted in Brazil and the nearby markets in the rest of Latin America.^[164–165]^

The pentavalent antimonials fall into a different category than the therapeutically active agents described in the earlier vignettes in one prominent way: we still do not know their molecular structures. As was highlighted throughout the vignettes, knowledge of molecular structure can play a critical role in the design of new and improved drugs. Without knowledge of the structures of the pentavalent antimonials, however, it has not been possible to systematically improve upon them, and they have gone unchanged for over 70 years. Moreover, lack of information about their structures makes quality assurance difficult, which has served as a historical source of complication for a number of the antimonial drugs, as described above. We propose that one of the complicating features of these substances has been their resistance to crystallization. We note that although crystallization could facilitate diffraction-based structure elucidation, it would more importantly provide a means of consistently obtaining a material of well-defined composition, which could then be interrogated using a range of analytical techniques. As technological developments continue, previously intractable structures are becoming increasingly accessible. One striking example lies in bismuth subsalicylate, an over-the-counter medication that is widely used to treat dyspepsia. This drug was in clinical use for well over a century before its structure was finally determined using three-dimensional electron diffraction (microED) techniques in 2022.^[166]^ Although bismuth subsalicylate does not suffer from the negative side effects that accompany treatment with the pentavalent antimonials, this structural information could prove invaluable in the development of new Bi-based drugs, an area of ongoing investigation.^[167]^

In the absence of such a well-defined composition, the possibility remains open that the pentavalent antimonials, as they are currently clinically employed, fall into scenarios b, c, or d of Figure 1. These drugs evidently contain active substances, but it is possible that the active species might be contaminated by inactive species and have its activity underestimated (Figure 1b) or be a minor component of the mixture (Figure 1c). Given the high reactivity of labile Sb complexes, any impurities present are likely to be biologically active and could easily introduce off-target toxicity (Figure 1d). Establishing the structures of these drugs will provide a route toward developing separation/purification protocols or at the very least defining criteria for chemical purity, which could help to improve antimonial therapy.

## Data Availability

Data sharing is not applicable to this article as no new data were created or analyzed in this study.

## References

[1] Joly A, Compt. Rendu. Acad. Sci. 1892, 115, 1299–1301.

[2] Mangin L, Compt. Rendu. Acad. Sci. 1893, 116, 653–656.

[3] Nicolle M, Cantacuzène J, Ann. Inst. Pasteur 1893, 7, 331–334.

[4] Luft JH, Anat. Rec. 1971, 171, 347–368. 4108333 10.1002/ar.1091710302

[5] Luft JH, Anat. Rec. 1971, 171, 369–415. 4108334 10.1002/ar.1091710303

[6] Gleu K, Breuel W, Z. Anorg. Allg. Chem. 1938, 237, 350–358.

[7] Fletcher JM, Greenfield BF, Hardy CJ, Scargill D, Woodhead JL, J. Chem. Soc. 1961, 2000–2006.

[8] Jørgensen CK, Orgel LE, Mol. Phys. 1961, 4, 215–218.

[9] Earley JE, Fealey T, J. Chem. Soc. D 1971, 331.

[10] Earley JE, Fealey T, Inorg. Chem. 1973, 12, 323–327.

[11] Clausen CA III, Prados RA, Good ML, Inorg. Nucl. Chem. Lett. 1971, 7, 485–489.

[12] Smith PM, Fealey T, Earley JE, Silverton JV, Inorg. Chem. 1971, 10, 1943–1947.

[13] de C. T. Carrondo MAAF, Griffith WP, Hall JP, Skapski AC, Biochim. Biophys. Acta Gen. Subj. 1980, 627, 332–334. 10.1016/0304-4165(80)90464-x 6153278

[14] Bondareff W, Anat. Rec. 1967, 157, 527–535.

[15] Bondareff W, J. Neurosurg. 1970, 32, 145–151. 4189442 10.3171/jns.1970.32.2.0145

[16] Deluca HF, Engstrom GW, Proc. Natl. Acad. Sci. USA 1961, 47, 1744–1750. 13885269 10.1073/pnas.47.11.1744 PMC223205

[17] Vasington FD, Murphy JV, J. Biol. Chem. 1962, 237, 2670–2677. 13925019

[18] Carafoli E, Trends Biochem. Sci. 2003, 28, 175–181. 12713900 10.1016/S0968-0004(03)00053-7

[19] Moore CL, Biochem. Biophys. Res. Commun. 1971, 42, 298–305. 4250976 10.1016/0006-291x(71)90102-1

[20] Vasington FD, Gazzotti P, Tiozzo R, Carafoli E, Biochim. Biophys. Acta Bioenerg. 1972, 256, 43–54. 10.1016/0005-2728(72)90161-2 4257941

[21] Reed KC, Bygrave FL, Biochem. J. 1974, 140, 143–155. 4375957 10.1042/bj1400143 PMC1167986

[22] Reed KC, Bygrave FL, FEBS Lett. 1974, 46, 109–114. 4371774 10.1016/0014-5793(74)80346-7

[23] Kehrer JP, Park Y, Pharmacol J. Methods 1991, 25, 179–183. 10.1016/0160-5402(91)90008-s 1712417

[24] Broekemeier KM, Krebsbach RJ, Pfeiffer DR, Mol. Cell. Biochem. 1994, 139, 33–40. 7531818 10.1007/BF00944201

[25] Ying W-L, Emerson J, Clarke MJ, Sanadi DR, Biochemistry 1991, 30, 4949–4952. 2036363 10.1021/bi00234a016

[26] Emerson J, Clarke MJ, Ying W-L, Sanadi DR, J. Am. Chem. Soc. 1993, 115, 11799–11805.

[27] Matlib MA, Zhou Z, Knight S, Ahmed S, Choi KM, Krause-Bauer J, Phillips R, Altschuld R, Katsube Y, Sperelakis N, Bers DM, J. Biol. Chem. 1998, 273, 10223–10231. 9553073 10.1074/jbc.273.17.10223

[28] Baughman JM, Perocchi F, Girgis HS, Plovanich M, Belcher-Timme CA, Sancak Y, Bao XR, Strittmatter L, Goldberger O, Bogorad RL, Koteliansky V, Mootha VK, Nature 2011, 476, 341–345. 21685886 10.1038/nature10234 PMC3486726

[29] Oxenoid K, Dong Y, Cao C, Cui T, Sancak Y, Markhard AL, Grabarek Z, Kong L, Liu Z, Ouyang B, Cong Y, Mootha VK, Chou JJ, Nature 2016, 533, 269–273. 27135929 10.1038/nature17656 PMC4874835

[30] Cao C, Wang S, Cui T, Su X-C, Chou JJ, Proc. Natl. Acad. Sci. USA 2017, 114, E2846–E2851. 28325874 10.1073/pnas.1620316114 PMC5389301

[31] Woods JJ, Nemani N, Shanmughapriya S, Kumar A, Zhang M, Nathan SR, Thomas M, Carvalho E, Ramachandran K, Srikantan S, Stathopulos PB, Wilson JJ, Madesh M, Selective A, ACS Cent. Sci. 2019, 5, 153–166. 30693334 10.1021/acscentsci.8b00773 PMC6346394

[32] Nathan SR, Pino NW, Arduino DM, Perocchi F, MacMillan SN, Wilson JJ, Inorg. Chem. 2017, 56, 3123–3126. 28244741 10.1021/acs.inorgchem.6b03108

[33] Wilson JJ, Nathan SR, J. Visualized Exp. 2017, e56527. 10.3791/56527 PMC5755246 29155737

[34] Woods JJ, Spivey JA, Wilson JJ, Eur. J. Inorg. Chem. 2022, e202100995.

[35] Urgiles J, Nathan SR, MacMillan SN, Wilson JJ, Dalton Trans. 2017, 46, 14256–14263. 28994442 10.1039/c7dt03085a

[36] Novorolsky RJ, Nichols M, Kim JS, Pavlov EV, Woods JJ, Wilson JJ, Robertson GS, Cereb J. Blood Flow Metab. 2020, 40, 1172–1181. 10.1177/0271678X20908523 PMC7238378 32126877

[37] Woods JJ, Rodriguez MX, Tsai C-W, Tsai M-F, Wilson JJ, Chem. Commun. 2021, 57, 6161–6164. 10.1039/d1cc01623g PMC8240128 34042919

[38] Bigham NP, Huang Z, Spivey J, Woods JJ, MacMillan SN, Wilson JJ, Inorg. Chem. 2022, 61, 17299–17312. 36260092 10.1021/acs.inorgchem.2c02930 PMC11649380

[39] Woods JJ, Novorolsky RJ, Bigham NP, Robertson GS, Wilson JJ, RSC Chem. Biol. 2023, 4, 84–93. 36685255 10.1039/d2cb00189f PMC9811523

[40] Huang Z, Spivey JA, MacMillan SN, Wilson JJ, Inorg. Chem. Front. 2023, 10, 591–599. 40765837 10.1039/d2qi02183h PMC12323816

[41] Huang Z, MacMillan SN, Wilson JJ, Angew. Chem. Int. Ed. 2023, 62, e202214920. 10.1002/anie.202214920 PMC9892296 36515400

[42] Woods JJ, Wilson JJ, Curr. Opin. Chem. Biol. 2020, 55, 9–18. 31869674 10.1016/j.cbpa.2019.11.006

[43] Bigham NP, Wilson JJ, J. Am. Chem. Soc. 2023, 145, 9389–9409. 37078795 10.1021/jacs.3c01984

[44] Huang Z, Wilson JJ, ChemMedChem 2023, 18, e202300106. 37015871 10.1002/cmdc.202300106

[45] Mertens RT, Gukathasan S, Arojojoye AS, Olelewe C, Awuah SG, Chem. Rev. 2023, 123, 6612–6667. 37071737 10.1021/acs.chemrev.2c00649 PMC10317554

[46] Chrestien JA, De la méthode ïatraleptique. Croullebois & Crochard: Paris, 1811.

[47] Anonymous editors, Edinburgh Med. Surg. J. 1815, 11, 239–243.

[48] Koch R, Br. Med. J. 1890, 2, 380–383. 10.1136/bmj.2.1546.380 PMC2208055 20753110

[49] Bruck C, Glück A, Münch. Med. Wochenschr. 1913, 57–62.

[50] Feldt A, Dtsch. Med. Wochenschr. 1913, 39, 549–551.

[51] Feldt A, Ber. Klin. Wochenschr. 1917, 54, 1111–1114.

[52] Merck’s E Annual Report of Recent Advances in Pharmaceutical Chemistry and Therapeutics 1917–1921. E. Merk Chemical Works: Darmstadt, 1925.

[53] Dixon WE, Br. Med. J. 1925, 1, 813–815. 20772027 10.1136/bmj.1.3357.813 PMC2226387

[54] Feldt A, Klin. Wochenschr. 1926, 5, 299–301.

[55] Zeise WC, Ann. Pharm. 1834, 11, 1–10.

[56] Fordos MJ, Gélis A, J. Prakt. Chem. 1845, 35, 321–328.

[57] Moellgaard H, Br. Med. J. 1925, 1, 643–647. 20771993 10.1136/bmj.1.3353.643 PMC2226439

[58] McCluskey KL, Eichelberger L, J. Am. Chem. Soc. 1926, 48, 136–139.

[59] Parkin G, J. Chem. Educ. 2006, 83, 791–799.

[60] Mann FG, Wells AF, Purdie D, J. Chem. Soc. 1937, 1828–1836.

[61] Gibson CS, Krishnaswami KR, Curr. Sci. 1938, 7, 202–204.

[62] Faltens MO, Shirley DA, J. Chem. Phys. 1970, 53, 4249–4264.

[63] Brown K, Parish RV, McAuliffe CA, J. Am. Chem. Soc. 1981, 103, 4943–4945.

[64] Baggio RF, Baggio S, J. Inorg. Nucl. Chem. 1973, 35, 3191–3200.

[65] Ruben H, Zalkin A, Faltens MO, Templeton DH, Inorg. Chem. 1974, 13, 1836–1839.

[66] Bryce RA, Charnock JM, Pattrick RAD, Lennie AR, J. Phys. Chem. A 2003, 107, 2516–2523.

[67] Forestier J, Bull. Mem. Soc. Med. Hop. Paris 1929, 53, 323–327.

[68] Forestier J, Lancet 1932, 219, 441–444.

[69] Kean TA, Ulster Med. J. 1934, 3, 284–289. 20476023 PMC2478925

[70] Forestier J, Lancet 1934, 224, 646–648.

[71] Mazid MA, Razi MT, Sadler PJ, Greaves GN, Gurman SJ, Koch MHJ, Phillips JC, J. Chem. Soc. Chem. Commun. 1980, 1261–1263.

[72] Elder RC, Ludwig K, Cooper JN, Eidsness MK, J. Am. Chem. Soc. 1985, 107, 5024–5025.

[73] Sutton BM, McGusty E, Walz DT, DiMartino MJ, J. Med. Chem. 1972, 15, 1095–1098. 4654656 10.1021/jm00281a001

[74] Braid J, Br. Med. J. 1858, 54–1, 214–215.

[75] Braid J, Br. Med. J. 1858, 54–1, 135–135.

[76] Livingstone D, Br. Med. J. 1858, s4–1, 360–361.

[77] Decelles C, J. Chem. Educ. 1949, 26, 583–587.

[78] Béchamp A, Compts. Rend. 1860, 51, 356–360.

[79] Béchamp MA, Compts. Rend. 1863, 56, 1172–1175.

[80] Thomas HW, Br. Med. J. 1905, 1, 1140–1143. 20762118 10.1136/bmj.1.2317.1140 PMC2320665

[81] Breinl A, Todd JL, Br. Med. J. 1907, 1, 132–134. 20763023 10.1136/bmj.1.2403.132 PMC2356533

[82] Moore B, Nierenstein M, Todd JL, Biochem. J. 1907, 2, 300–324. 16742071 10.1042/bj0020300 PMC1276215

[83] Ehrlich P, Berl. Klin. Wochenschr. 1907, 44, 280–283.

[84] Ehrlich P, Bertheim A, Ber. Dtsch. Chem. Ges. 1907, 40, 3292–3297.

[85] Schaudinn FR, Hoffmann E, Arb. Kais. Gesundheitsamte 1905, 22, 527–534.

[86] Uhlenhuth P, Hoffmann E, Roscher K, Dtsch. Med. Wochenschr. 1907, 33, 873–876.

[87] Ehrlich P, Partial Cell Functions (Nobel Lecture). Nobel Media AB: 1908.

[88] Ehrlich P, Bertheim A, Ber. Dtsch. Chem. Ges. 1911, 44, 1260–1269.

[89] Ehrlich P, Bertheim A, Ber. Dtsch. Chern. Ges. 1912, 45, 756–766.

[90] Schweitzer H, Science 1910, 32, 809–823. 17750500 10.1126/science.32.832.809

[91] Cowley AH, Lasch JG, Norman NC, Pakulski M, J. Am. Chem. Soc. 1983, 105, 5506–5507.

[92] Burns JH, Waser J, J. Am. Chem. Soc. 1957, 79, 859–864.

[93] Hedberg K, Hughes EW, Waser J, Acta Crystallogr. 1961, 14, 369–374.

[94] Badger GM, Drewer RJ, Lewis GE, Aust. J. Chem. 1963, 16, 285–288.

[95] Levinson AS, J. Chem. Educ. 1977, 54, 98–99.

[96] Lloyd NC, Morgan HW, Nicholson BK, Ronimus RS, Angew. Chem. Int. Ed. 2005, 44, 941–944. 10.1002/anie.200461471 15624113

[97] Friedheim EAH, Ann. Inst. Pasteur 1940, 65, 108–118.

[98] Friedheim EAH, J. Am. Chem. Soc. 1944, 66, 1775–1778.

[99] Friedheim EAH, Ann. Trop. Med. Parasitol. 1948, 42, 357–363. 18110349 10.1080/00034983.1948.11685383

[100] Lindquist-Kleissler B, Wenger JS, Johnstone TC, Inorg. Chem. 2021, 60, 1846–1856. 33471517 10.1021/acs.inorgchem.0c03308

[101] Wenger JS, Johnstone TC, Chem. Commun. 2021, 57, 3484–3487. 10.1039/d1cc00619c 33688905

[102] Wenger JS, Wang X, Johnstone TC, Inorg. Chem. 2021, 60, 16048–16052. 34661394 10.1021/acs.inorgchem.1c02229

[103] Wenger JS, Weng M, George GN, Johnstone TC, Nat. Chem. 2023, 15, 633–640. 36959510 10.1038/s41557-023-01160-x PMC10159848

[104] Institute of Medicine (U.S.). Committee to Survey the Health Effects of Mustard Gas and Lewisite, Veterans at Risk : the health effects of mustard gas and Lewisite. National Academy Press: Washington, D. C., 1993, xviii, 427 p.

[105] Peters RA, Stocken LA, Thompson RHS, Nature 1945, 156, 616–619. 21006485 10.1038/156616a0

[106] Waters LL, Stock C, Science 1945, 102, 601–606. 10.1126/science.102.2659.601 17818184

[107] Cohen A, King H, Strangeways WI, J. Chem. Soc. 1931, 3043–3057.

[108] Friedheim EAH, Ann. Trop. Med. Parasitol. 1959, 53, 1–9. 13650482 10.1080/00034983.1959.11685892

[109] Friedheim EAH, Am. J. Trop. Med. Hyg. 1949, s1–29, 173–180. 10.4269/ajtmh.1949.s1-29.173 18116843

[110] Kuepfer I, Schmid C, Allan M, Edielu A, Haary EP, Kakembo A, Kibona S, Blum J, Burri C, PLoS Neglected Trop. Dis. 2012, 6, e1695. 10.1371/journal.pntd.0001695 PMC3435133 22970329

[111] WHO interim guidelines for the treatment of gambiense human African trypanosomiasis. World Health Organization: Geneva, 2019. 31449367

[112] Plimmer HG, Thomson JD, Proc. R. Soc. London Ser. B 1907, 79, 505–516.

[113] Plimmer HG, Thomson JD, Proc. R. Soc. London Ser. B 1908, 80, 1–10.

[114] Plimmer HG, Bateman HR, Proc. R. Soc. London Ser. B 1908, 80, 477–487.

[115] Plimmer HG, Fry WB, Proc. R. Soc. London Ser. B 1909, 81, 354–371.

[116] Thomson JD, Cushny AR, Proc. R. Soc. London Ser. B 1910, 82, 249–256.

[117] Dumas J, Piria R, Justus Liebigs Ann. Chem. 1842, 44, 66–100.

[118] Plimmer RHA, Proc. R. Soc. London Ser. B 1908, 80, 11–12.

[119] Duffin J, René P, J. Hist. Med. Allied Sci. 1991, 46, 440–456. 1744431 10.1093/jhmas/46.4.440

[120] Kamenar B, Grdnić D, Prout CK, Acta Crystallogr. Sect. B 1970, 26, 181–188. 5467310 10.1107/s0567740870002133

[121] Leishman WB, Br. Med. J. 1903, 1, 1252–1254.

[122] Donovan C, Br. Med. J. 1903, 2, 79.

[123] Donovan C, Br. Med. J. 1903, 2, 1401.20761210

[124] Leishman WB, Br. Med. J. 1903, 2, 1376–1377.

[125] Ross R, Br. Med. J. 1903, 2, 1261–1262. 20761169 10.1136/bmj.2.2237.1261 PMC2514667

[126] Ross R, Br. Med. J. 1903, 2, 1401.20761210

[127] Donovan C, Indian Med. Gaz. 1903, 38, 478. PMC5150752 29002982

[128] Gibson ME, Med. Hist. 1983, 27, 203–213. 6345968 10.1017/s0025727300042691 PMC1139308

[129] Carini A, Paranhos U, Bull. Soc. Pathol. Exot. Ses Fil. 1909, 2, 255–257.

[130] Lindenberg A, Bull. Soc. Pathol. Exot. Ses Fil. 1909, 2, 252–254.

[131] Manson P, Trans. R. Soc. Trop. Med. Hyg. 1908, 1, 126–130.

[132] M’Kaig A, Edinburgh Med. J. 1908, 1, 539–540.

[133] Brahmachari UN, Br. Med. J. 1908, 1, 1286–1288. 20763864 10.1136/bmj.1.2474.1286 PMC2436723

[134] Da Silva P, Arch. Parasitol. 1911, 15, 401–424.

[135] Darling ST, Arch. Intern. Med. 1911, 7, 581–597.

[136] Aragăo HDB, Vianna G, Mem. Inst. Oswaldo Cruz 1913, 5, 211–238.

[137] di Cristina G, Caronia G, Dtsch. Med. Wochenschr. 1915, 41, 396–397.

[138] Rogers L, Br. Med. J. 1915, 2, 197.

[139] Breinl A, Nierenstein M, Ann. Trop. Med. Parasitol. 1909, 2, 365–382.

[140] Carmalt CJ, Crossley JG, Norman NC, Orpen AG, Chem. Commun. 1996, 1675–1676.

[141] Bordner J, Doak GO, Everett TS, J. Am. Chem. Soc. 1986, 108, 4206–4213.

[142] Wenger JS, Getahun A, Johnstone TC, Dalton Trans. 2023, 52, 11325–11334. 37530432 10.1039/d3dt02113k

[143] Beckmann J, Finke P, Hesse M, Wettig B, Angew. Chem. Int. Ed. 2008, 47, 9982–9984. 10.1002/anie.200803997 19006136

[144] Nicholson BK, Clark CJ, Wright CE, Groutso T, Organometallics 2010, 29, 6518–6526.

[145] Beckmann J, Hesse M, Main Group Met. Chem. 2013, 36, 141–143.

[146] Liu Z-q., Ozawa Y, Yagasaki A, Bull. Chem. Soc. Jpn. 2014, 87, 1245–1251.

[147] Zaitseva EG, Medvedev SV, Aslanov LA, J. Struct. Chem. 1990, 31, 261–267.

[148] Brahmachari UN, Indian J Med. Res. 1922, 10, 492–522. 2644172

[149] Shortt HE, Sen RT, Indian J Med. Res. 1923, 11, 653–659.

[150] Brahmachari UN, Das J, Indian J Med. Res. 1924, 12, 423–426.

[151] Brahmachari UN, Indian J Med. Res. 1925, 13, 111–112.

[152] Brahmachari UN, Chapter14. Treatment. In Treatise on Kala-Azar, John Bale, Sons & Danielsson, Ltd.: London, 1928, pp. 110–145.

[153] Napier LE, Indian Med. Gaz. 1925, 60, 24–26. PMC5189086 29010427

[154] Struthers EB, Chin. Med. J. 1927, 41, 21–28.

[155] Megaw JWD, Indian Med. Gaz. 1926, 61, 291–293. PMC5232245 29011297

[156] Gray WH, Trevan JW, Bainbridge HW, Attwood AP, Proc. R. Soc. London Ser. B 1931, 108, 54–83.

[157] Brahmachari U, Nature 1940, 145, 1021–1022.

[158] Guha RC, Dutta NK, Mukerji B, Nature 1943, 151, 108–109.

[159] Ghosh S, Chopra RN, Chatterjee NR, Indian J Med. Res. 1928, 16, 461–468.

[160] Brahmachari PN, Sir Upendranath Brahmachari. In Biographical Memoirs of Fellows of the Indian National Science Academy, Indian National Science Academy: New Delhi, 1976, Vol. 4.

[161] Kikuth W, Schmidt H, Chin. Med. J. 1937, 52, 425–432.

[162] Thompson RB, Lancet 1944, 243, 17–18.

[163] Goodwin LG, Trans. R. Soc. Trop. Med. Hyg. 1995, 89, 339–341. 7660456 10.1016/0035-9203(95)90572-3

[164] Barbeitas MM, La recherche et le développement pharmaceutique pour les maladies négligées sous l’angle de la leishmaniose: rupture ou continuité dans la santé globale? PhD Dissertation, École des Hautes Etudes en Sciences Sociales, Paris, 2022.

[165] Carvalho SH, Frézard F, Pereira NP, Moura AS, Ramos LMQC, Carvalho GB, Rocha MOC, Trop. Med. Int. Health 2019, 24, 380–391. 30681239 10.1111/tmi.13210

[166] Svensson Grape E, Rooth V, Nero M, Willhammar T, Inge AK, Nat. Commun. 2022, 13, 1984. 35418171 10.1038/s41467-022-29566-0 PMC9008038

[167] Griffith DM, Li H, Werrett MV, Andrews PC, Sun H, Chem. Soc. Rev. 2021, 50, 12037–12069. 34533144 10.1039/d0cs00031k

[168] Benedek TG, J. Hist. Med. Allied Sci. 2004, 59, 50–89. 15011812 10.1093/jhmas/jrg042

